# Early versus Late Radiofrequency Catheter Ablation in Atrial Fibrillation: Timing Matters

**DOI:** 10.3390/jcm13164643

**Published:** 2024-08-08

**Authors:** Ahmad A. A. Farghaly, Hussam Ali, Pierpaolo Lupo, Sara Foresti, Guido De Ambroggi, Salah Atta, Ahmed Abdel-Galeel, Aly Tohamy, Riccardo Cappato

**Affiliations:** 1 Arrhythmia & Electrophysiology Center, IRCCS MultiMedica, Sesto San Giovanni, 20099 Milan, Italy; ahmadfarghaly@med.aun.edu.eg (A.A.A.F.); pierpaolo.lupo@multimedica.it (P.L.); sara.foresti@multimedica.it (S.F.); guido.deambroggi@multimedica.it (G.D.A.); riccardo.cappato@multimedica.it (R.C.); 2 Department of Cardiovascular Medicine, Assiut University Heart Hospital, Faculty of Medicine, Assiut University, Assiut 71526, Egypt; salah_s_atta@hotmail.com (S.A.); ahmed.galeel@aun.edu.eg (A.A.-G.); ali.tohamy@gmail.com (A.T.)

**Keywords:** atrial fibrillation, radiofrequency catheter ablation, diagnosis-to-ablation time, atrial arrhythmia recurrence, cardiovascular hospitalizations, AF progression

## Abstract

**Background:** Despite the progressive course of atrial fibrillation (AF), the optimal timing of radiofrequency catheter ablation (RFCA) during disease course is still unknown. We aimed to investigate the impact of early RFCA within a year after AF diagnosis on procedural outcomes. **Methods:** A single-center retrospective study was conducted on symptomatic AF patients (*n* = 130) referred for RFCA with a 16-month median follow-up. Patients were stratified based on the diagnosis-to-ablation time (DAT) into early (≤1 year) and late (>1 year) RFCA groups. Atrial arrhythmia recurrence after single RFCA was the primary outcome. Secondary outcomes included cardiovascular hospitalizations, AF progression, and antiarrhythmic drug (AAD) use. **Results:** Within a year of AF diagnosis, 33 patients (25.4%) underwent RFCA. In the early-RFCA group, 84.4% of patients did not have recurrent atrial arrhythmia, in contrast to 60.8% in the late-RFCA group (*p* = 0.039). Late RFCA (HR = 2.74, 95% CI = 1.062–7.052, *p* = 0.037) and AF recurrence during the blanking period (HR = 4.57, 95% CI = 2.38–8.57, *p* < 0.0001) were independent predictors of atrial arrhythmia recurrence on multivariate analysis. Compared to the late-RFCA group, the early-RFCA group had significantly lower rates of cardiovascular hospitalizations (18% vs. 42%, *p* = 0.023), AF progression (0.0% vs. 11.3%, *p* = 0.044), and AAD use (45.4% vs. 81.4%, *p* < 0.001). **Conclusions:** Early RFCA within a year of AF diagnosis is associated with less atrial arrhythmia recurrence, fewer cardiovascular hospitalizations, less AF progression, and less AAD use. DAT of more than one year and AF recurrence during the blanking period are independent predictors of atrial arrhythmia recurrence after single RFCA.

## 1. Introduction

Catheter ablation (CA) is an effective strategy for symptomatic atrial fibrillation (AF) patients, reducing recurrent atrial arrhythmias and cardiovascular hospitalizations [1,2]. However, reported ablation outcomes are still unsatisfactory, with rates of atrial arrhythmia recurrence varying between 20% to 50% at one year post-ablation [3,4].

During the natural course of the disease, structural and electrical remodeling evolve over time and trigger atrial fibrosis, which initiates a vicious cycle of remodeling and promotes AF progression and adversely affects the ablation outcomes [5,6,7].

According to the recent AF guidelines of the European Society of Cardiology (ESC 2020), CA is recommended (Class I indication) for symptomatic paroxysmal/persistent AF refractory to or intolerant of at least one class I or III antiarrhythmic drug (AAD) [8]. Moreover, CA should be considered (Class IIa indication) as first-line rhythm control therapy (without prior AAD use) in selected patients with symptomatic paroxysmal AF [8]. However, the optimal timing for CA during the disease course is not yet elucidated.

Prior studies have investigated the impact of CA timing on clinical outcomes [2,9,10,11]. However, the data were contradictory. In our study, we aimed to assess the impact of early radiofrequency CA (RFCA) within one year after AF diagnosis on procedural outcomes, hypothesizing that early RFCA would obviate the vicious cycle of disease progression and improve procedural outcomes.

## 2. Materials and Methods

### 2.1. Study Populations

This was a single-center retrospective study. The study enrolled symptomatic AF patients who were intolerant to or had failed AAD referred to our center for RFCA between September 2020 and September 2022 at IRCCS Multimedica hospital, Sesto San Giovanni, Milan, Italy. The follow-up was finished in March 2023. AF diagnosis was established by electrocardiographic documentation of AF, captured on a standard 12-lead ECG, or lasting at least 30 s on Holter ECG recordings [8]. Diagnosis-to-ablation time (DAT) was defined as the time interval between the first documented AF episode and the ablation procedure. Though arbitrary, a 12-month period was considered a reasonable cutoff for the early-ablation group, allowing for a comprehensive clinical assessment of AF patients, their comorbidities, AAD efficacy, and the arrhythmic burden. Patients were stratified based on the DAT into two groups: early-RFCA group (DAT ≤ one year) and late-RFCA group (DAT > one year). Eligible patients had paroxysmal or persistent symptomatic AF with ≥2 AF episodes over the 6 months before ablation (AF episode defined as ≥30 s). Exclusion criteria were (i) previous AF ablation, (ii) long-lasting persistent or permanent AF, (iii) inability to consent, and (iv) age < 18 years old.

### 2.2. RFCA Protocol

All clinical characteristics (age, sex, and body mass index (BMI)) were addressed. AF type, EHRA class, previous use of rate-control drugs (beta-blockers, calcium channel blockers, and digoxin), and/or rhythm-control drugs (class I, III AAD), and history of electrical cardioversion were reported. History of comorbidities was mentioned. CHA2DS2VASc, CHADS2, and HAS-BLEED scores were calculated for all patients. Trans-thoracic echocardiography was carried out within a week before the procedures to report the left atrial (LA) diameter and left ventricular ejection fraction (LV EF). Transesophageal echocardiography was performed within 24 h before the procedure to exclude the presence of LA thrombi. AADs were stopped 4 to 5 half-lives before ablation. In patients on vitamin K antagonists, ablation was performed under therapeutic INR values of 2 to 3. Direct oral anticoagulants were stopped on the morning of the procedure.

The procedure was performed under general anesthesia. A quadripolar and a decapolar catheter were placed at the His bundle and into the coronary sinus, respectively. Following transseptal puncture, two long sheaths (8.5 F) were advanced into the LA, and then unfractionated heparin was administered to maintain an activated clotting time of 250–300 s during the procedure. Pulmonary venography was performed by injecting contrast during rapid RV pacing. A circular mapping catheter (Lasso 25–15 catheter) was used to record the pulmonary vein potentials. Ablation was performed by elimination of all potentials at the ostium of the pulmonary veins using a 3.5 mm irrigated catheter (ThermoCool SF Bi-Directional Catheter, Biosense Webster, Inc., Irvine, CA, USA).

Successful pulmonary vein isolation (PVI) was defined as complete elimination or dissociation of potentials inside the pulmonary veins guided by the mapping catheter [8]. In patients who were still in AF post-PVI, electrical cardioversion to sinus rhythm was carried out and followed by PVI confirmation. For patients who had documented or induced typical atrial flutter (AFL), a cavo-tricuspid isthmus (CTI) line was placed and followed by confirmation of bidirectional block as an endpoint. Total procedure time (minutes), fluoroscopy time (minutes) and RF duration (minutes) were recorded in the two groups.

During hospitalization post-ablation, a complete clinical examination of all patients was carried out to detect procedural complications. Telemetry and ECG were performed to detect any arrhythmia. Transthoracic echocardiograms were routinely taken to exclude pericardial effusion. Anticoagulation was continued for at least 3 months and stopped thereafter based on the individual CHA2DS2-VASc score. AADs were continued for 3 months and stopped thereafter at the discretion of the referring physician. Patients were discharged one day after the procedure.

### 2.3. Follow-Up Management

All patients had scheduled outpatient visits at 3, 6, and 12 months post-ablation and then 6 months thereafter with 12-lead ECG and Holter (24–72 h). Between scheduled visits and in cases of symptoms, patients were encouraged to seek the nearest medical facility to document any arrhythmia. Interrogation of implanted devices (pacemakers or implantable loop recorders (ILRs)) was undertaken to assess recurrences.

### 2.4. Study Endpoints

The primary effectiveness endpoint was freedom from any atrial arrhythmia (atrial arrhythmia, defined as AF, AFL, or atrial tachycardia (AT) that lasted ≥30 s on/off AAD detected by the monitoring tools during the follow-up period excluding the first three months of the blanking period).

The secondary effectiveness endpoints were cardiovascular hospitalization, AF progression and AAD use during the follow-up period.

Cardiovascular hospitalizations were defined as any hospitalizations due to cardiovascular cause, such as AF-related hospitalizations (such as emergency department (ED) visits, repeat ablation, electrical cardioversion and AV nodal ablation), device implantations, and decompensated HF hospitalization, stroke, myocardial infarction, vascular complications and major bleedings.

AF progression was defined as progression from paroxysmal to persistent/permanent AF or persistent to permanent AF [5]. AAD use was defined as AAD use after the blanking period.

The safety endpoint was the absence of procedural complications, including vascular access-related complications (hematomas, femoral artery pseudoaneurysm, arteriovenous fistula), pericardial effusion, cardiac tamponade, pericarditis, transient ischemic attack, embolic stroke, PV stenosis, atrioesophageal fistula, myocardial infarction, and death.

### 2.5. Statistical Methods

Categorical variables are summarized as frequencies and percentages. Continuous variables are expressed in terms of means ± standard deviation (SD) if normally distributed or median (interquartile range) if abnormally distributed. Differences in variables between groups were analyzed using chi-squared/Fisher’s exact test for categorical variables and Z-test or McNemar test for continuous variables. Kaplan–Meier survival analysis was performed using multiple-direction log-rank tests [12]. In the multivariate regression analysis, we included predefined variables, which were age, DAT > 1 year, persistent AF type, LA diameter > 50 mm, and AF recurrence during the blanking period. Selection of the variables based on clinical and published data [13,14,15,16]. The adjusted hazard function was estimated using nonparametric smoothing [17]. The confidence interval was 95%, and a *p*-value ≤ 0.05 was considered statistically significant. All statistical analysis was performed using R-Cran software version 4.3.0. (R Foundation for Statistical Computing, Vienna, Austria).

## 3. Results

### 3.1. Baseline Characteristics (Table 1)

We identified 130 symptomatic AF patients who underwent the first RFCA. The mean patient age was 59.2 ± 10.7 years, and 24.6% of patients were female. Thirty-four (26.2%) patients had persistent AF. Structural heart diseases (SHD) were present in 26 (20%) patients. Thirty-three (25.4%) patients had the ablation procedure performed within one year of documented AF diagnosis. For the entire study group, the mean DAT of the whole cohort was 42.99 ± 4.3 months. Mean DAT was 7.6 ± 1.3 months in the early-RFCA group and 55.02 ± 5.2 months in the late-RFCA group. Baseline characteristics were similar in both groups, apart from previous attempts at rhythm control, either by class I, III AADs or by electrical cardioversion (ECV), which more frequently performed in late-RFCA group (82.5% vs. 66.4%, *p* 0.033 and 52.1% vs. 36.4%, *p* 0.049, respectively). Median follow-up duration was 16 months (IQR: 10–21).

**Table 1:** Baseline characteristics.

	Total (*n* = 130)	Early RFCA (*n* = 33)	Late RFCA (*n* = 97)	*p*-Value
Age (years)	59.15 ± 10.7	57.30 ± 12.7	59.77 ± 9.9	0.256
Diagnosis-to-ablation time (months)	42.99 ± 4.3	7.64 ± 1.3	55.02 ± 5.2	<0.001
Sex				
• Female	32 (24.6%)	10 (30.3%)	22 (22.7%)	0.380
BMI	26.72 ± 3.6	25.87 ± 4.1	27.03 ± 3.4	0.164
Blood Pressure				
• SBP	126.98 ± 11.7	125.76 ± 10.8	128.83 ± 13.6	0.201
• DBP	76.04 ± 12.2	74.48 ± 13.6	77.63 ± 11.4	0.242
GFR	86.75 ± 15.9	89.42 ± 16.7	84.44 ± 15.2	0.116
Comorbidity				
• DM	5 (3.8%)	3 (9.1%)	2 (2.1%)	0.103
• HTN	71 (54.6%)	16 (48.5%)	55 (56.7%)	0.413
• IHD	10 (7.7%)	1 (3%)	9 (9.3%)	0.224
• History of CHF	7 (5.4%)	3 (9.1%)	4 (4.2%)	0.273
• SHD	27 (20%)	5 (18.2%)	21 (20.6%)	0.762
• OSAS	4 (3.1%)	1 (3%)	3 (3.1%)	0.733
• History of stroke	9 (7%)	2 (6.1%)	7 (7.3%)	0.584
AF type				
• PAF	96 (73.8%)	26 (78.8%)	70 (72.2%)	0.455
CHA2DS2-VASc score				
• 0	36 (27.7%)	11 (33.3%)	25 (25.8%)	
• 1	36 (27.7%)	7 (21.2%)	29 (29.9%)	
• 2	34 (26.2%)	9 (27.3%)	25 (25.8%)	0.472
• 3	20 (15.4%)	4 (12.1%)	16 (16.5%)	
• 4	4 (3.1%)	2 (6.1%)	2 (2.1%)	
✓ Mean ± SD	1.38 ± 1.1	1.36 ± 1.2	1.39 ± 1.1	0.909
CHADS2 score				
• 0	50 (38.5%)	15 (45.5%)	35 (36.1%%	
• 1	64 (49.2%)	11 (33.3%)	53 (54.6%)	0.112
• 2	9 (6.9%)	4 (12.1%)	5 (5.2%)	
• 3	7 (5.4%)	3 (9.1%)	4 (4.1%)	
✓ Mean ± SD	0.79 ± 0.79	0.85 ± 0.97	0.77 ± 0.97	0.932
HAS-BLED score				
• 0	68 (52.3%)	20 (60.6%)	48 (49.5%)	
• 1	51 (39.2%)	10 (30.3%)	41 (42.3%)	0.245
• 2	11 (8.5%)	3 (9.1%)	8 (8.2%)	
✓ Mean ± SD	0.56 ± 0.6	0.48 ± 0.7	0.59 ± 0.6	0.444
LA Diameter	39.66 ± 3.9	39.33 ± 3.8	40.44 ± 4.1	0.198
LVEF%	60.75 ± 15.9	59.05 ± 5.8	60.52 ± 4.2	0.127
Beta-blocker use	46 (35.4%)	11 (33.3%)	35 (36.1%)	0.775
CCB use	16 (12.3%)	3 (9.1%)	13 (13.4%)	0.518
Class I, III AAD use	105 (80.7%)	23 (69.7%)	82 (84.5%)	0.033
ACEI use	69 (53.1%)	15 (45.5%)	54 (55.7%)	0.312
Statin use	30 (23.1%)	6 (18.2%)	24 (24.7%)	0.479
MRA	3 (2.31%)	1 (3.03%)	2 (2.02%)	0.738
Previous electrical CV	62 (48.1%)	12 (36.4%)	50 (52.1%)	0.049
Follow-up duration	16.32 ± 6.632	16.39 ± 6.324	16.21 ± 6.622	0.975

AAD, antiarrhythmic drug; ACEI, angiotensin-converting enzyme inhibitor; BMI, body mass index; CCB, calcium channel blocker; CHF, congestive heart failure; CV, cardioversion; DBP, diastolic blood pressure; DM, diabetes mellitus; GFR, glomerular filtration rate; HTN, hypertension; LA, left atrium; LVEF, left ventricular ejection fraction; IHD, ischemic heart disease; MRA, mineralocorticoid receptor agonist; OSAS, obstructive sleep apnea; PAF, paroxysmal atrial fibrillation; SBP, systolic blood pressure; SHD, structural heart disease.

### 3.2. Procedural Characteristics

Successful PVI was achieved in all patients. CTI ablation was performed in nine (6.9%) patients with documented atrial flutter. Total RF and fluoroscopy times were significantly lower in the early-RFCA group than in the late-RFCA group (25.38 ± 6.4 vs. 30.56 ± 5.1, *p* 0.012, 35.88 ± 3.5 vs. 44.48 ± 2.2, *p* 0.031, respectively).

### 3.3. Follow-Up Rhythm Monitoring (Table 2)

Post-ablation rhythm monitoring was performed with intermittent monitoring (Holter 24–72 h) in the majority of patients (90.8%) and with continuous cardiac monitoring (including ILR or permanent pacemaker) in the rest of the patients (9.2%). The proportion of ICM and Holter monitoring did not show significant differences between the groups.

**Table 2:** Follow-up rhythm monitoring.

	Total (*n* = 130)	Early RFCA (*n* = 33)	Late RFCA (*n* = 97)	*p*-Value
• Holter	118 (90.8%)	30 (90.9%)	88 (90.7)	=0.822
• ICM	12 (9.2%)	3 (9.1%)	9 (9.3%)	

ICM, implantable cardiac monitoring.

### 3.4. Outcomes

#### 3.4.1. Primary Outcome

**Figure 1:**
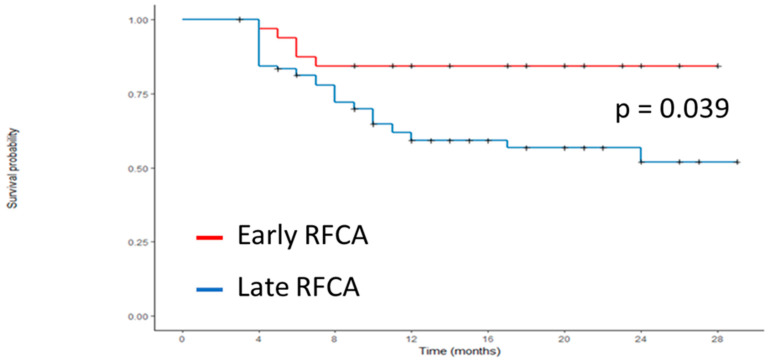
Arrhythmia-free survival after RFAC between early- and late-RFCA groups. RFCA, radiofrequency catheter ablation.

Documented atrial arrhythmia occurred in 43 patients (33.11%) of the whole cohort. The atrial arrhythmia-free rate was significantly higher in the early-RFCA group than in the late-RFCA group (84.8% vs. 60.8%, *p* 0.039) (Figure 1).

##### Predictors of Atrial Arrhythmia Recurrence (Table 3)

Using univariate Cox regression analysis, atrial arrhythmia recurrence was significantly associated with age ≥ 65 years, DAT > 1 year, persistent AF, CHADS2VASC score ≥ 2 and AF recurrence during the blanking period. On multivariate analysis, late RFCA (DAT > 1 year) (HR 2.74, 95% CI 1.062–7.052, *p* 0.037) and AF recurrence during the blanking period (HR 4.57, 95% CI 2.38–8.57, *p* 0.0001) were independent predictors of atrial arrhythmia recurrence after a single AF ablation procedure.

**Table 3:** Cox regression analysis for prediction of atrial arrhythmia recurrence.

	Univariate Analysis			Multivariate Analysis		
Predictors	HR	95% CI	*p* Value	HR	95% CI	*p* Value
Age ≥ 65	2.238	1.23–4.071	0.008	1.582	0.696–3.616	0.296
DAT (>1 year)	3.001	1.182–7.632	0.021	2.737	1.062–7.052	0.037
Sex (Female)	1.359	0.694–2.659	0.372			
BMI	0.994	0.854–1.104	0.998			
Persistent AF	2.181	1.182–4.022	0.012	1.373	0.649–2.902	0.407
Hypertension	1.139	0.623–2.08	0.672			
DM	1.142	0.764–1.042	0.445			
Ischemic heart disease	0.493	0.119–2.045	0.332			
Heart failure	0.981	0.237–4.061	0.984			
Left ventricular hypertrophy	0.796	0.245–2.579	0.704			
CHADS score ≥ 2	1.663	0.73–3.74	0.219			
CHA2DS2VASC score ≥ 2	2.251	1.223–4.153	0.001	1.399	0.628–7.133	0.411
Left atrial diameter ≥ 50 mm	2.875	0.887–9.318	0.08	1.268	0.359–4.607	0.699
LVEF < 50%	0.988	0.135–7.202	0.991			
AF recurrence during blanking	4.592	2.491–8.466	<0.01	4.517	2.380–8.571	<0.0001

CI, confidence interval; HR, hazard ratio.

#### 3.4.2. Secondary Outcomes (Table 4)

Compared to the late-RFCA group, the early-RFCA group had significantly fewer all cardiovascular hospitalizations (18.2% vs. 42.3%, *p* 0.023) and lower AAD use (39.4% vs. 77.3%, *p* < 0.001) after a median 16 months of follow-up. AF progression occurred in 11 patients (8.4%) and was observed only in the late-RFCA group. AF progressed from paroxysmal to persistent AF in ten patients, and one patient had progression from persistent to permanent AF. Late RFCA showed a significantly higher rate of AF progression compared to early RFCA (11.3% vs. 0%, *p* 0.044). No differences were observed in electrical cardioversion (4% vs. 9.3%, *p* 0.89), emergency department AF-related visits (0.0% vs. 8.2%, *p* 0.19), or repeat ablation (6.1% vs. 19.6%, *p* 0.12) between the groups.

**Table 4:** Secondary outcomes.

	Total (*n* = 130)	Early RFCA (*n* = 33)	Late RFCA (*n* = 97)	*p*-Value
All cardiovascular hospitalizations	47 (36.2%)	6 (18.2%)	41 (42.3%)	=0.023
Repeat ablation	21 (16.2%)	2 (6.1%)	19 (19.6%)	=0.123
Redo electrical cardioversion	13 (10%)	4 (12.1%)	9 (9.3%)	=0.891
Emergency department AF hospitalizations	8 (6.2%)	0 (0%)	8 (8.2%)	=0.199
AF-related hospitalizations	42 (32.3%)	6 (18.2%)	36 (37.1%)	=0.071
Non-AF-related hospitalizations Pacemaker for bradycardia, 2 cases of HF, PVC ablation and major bleeding	5 (3.1%)	0 (0%)	5 (4.1%)	=0.582
AAD use	94 (72.3%)	15 (45.4)	79 (81.4)	<0.001
AF progression	11 (8.5%)	0 (0%)	11 (11.3%)	=0.044

PVC, premature ventricular contraction.

#### 3.4.3. Safety Outcomes

Two arteriovenous fistulae were reported in 2 patients (1.5%, one complication in each group). Both complications were managed conservatively and did not require surgical intervention, but the hospital stay had been prolonged. There were no serious complications, as there were no cardiac tamponades or atrioesophageal fistulae detected.

## 4. Discussion

The present study complements the current body of evidence by assessing the influence of DAT on atrial arrhythmia recurrence, AF progression, and hard clinical outcomes such as cardiovascular hospitalizations. A real-world cohort of patients was enrolled and included both paroxysmal and persistent AF types in the presence or absence of structural heart diseases. Furthermore, to mitigate the influence of selection bias on the ablation timing reported in the previous retrospective studies [18,19,20,21], which was left to the discretion of the referring physician, the current study included only symptomatic AF patients with two or more AF episodes during the last six months before ablation.

The main findings are as follows. (I) Early RFCA within one year after the first documented AF is associated with less atrial arrhythmia recurrence, cardiovascular hospitalizations, AF progression, and AAD use. (II) Longer DAT (>one year) and AF recurrence during the blanking period are the only independent predictors of atrial tachyarrhythmia recurrence after single RFCA.

### 4.1. Early RFCA and Atrial Arrhythmia Recurrence

Early RFCA was associated with significantly lower incidence of arrhythmia recurrence, consistent with prior retrospective studies [18,19,20,21]. Assessment of RFCA timing has been studied previously by Kawaji et al. [18], where patients were divided into two groups based on DAT: short (≤3 years) DAT (*n* = 675) and long (≥3 years) DAT (*n* = 531) with a 5-year mean follow-up. Radiofrequency PVI and CTI ablation were routinely performed for all patients. Notably, the long-DAT-group patients had more history of CHF and stroke. In our study, one year was used as a cutoff to define the early-RFCA group. Patients had comparable baseline characteristics (age), risk factors (CHF and stroke), and AF types in both groups. Additionally, CTI ablation was performed only in patients who had clinically documented or induced atrial flutter.

The impact of DAT on AF recurrence has been recently investigated to demonstrate the upper and lower DAT thresholds [19]. De Greef et al. recommended performing CA within 3 years after first AF diagnosis, where the shorter the DAT (≤3 years), the better the outcome. However, when DAT exceeds three years, it becomes clinically irrelevant. According to De Greef’s study, all patients had scheduled visits every six months (only physical examination and ECG were performed). Also, unscheduled visits were conducted if symptoms occurred. In cases of symptoms, ECG, Holter monitoring (1 to 7 days), or event recording was used to report any related arrhythmia. Therefore, the follow-up assessment of asymptomatic recurrences was undetected. In our study, Holter monitoring (1–3 days) was performed in 90% of patients at 3, 6, 12 months, and then every 6 months during follow-up. Continuous cardiac monitoring (cardiac pacemakers and ILR) was used in approximately 10% of our patients. Thus, more precise detection of both asymptomatic and symptomatic recurrent atrial arrhythmia was possible by the use of different monitoring strategies in our study.

In a newly published prospective, randomized, multicenter study of symptomatic paroxysmal and persistent AF patients, an early-ablation strategy (within 1 month post-recruitment) was compared to a delayed ablation strategy (after 12 months post-recruitment, during which AADs were optimized) [22]. Both strategies had similar arrhythmia-free survival, median AF burden, and AAD use at 12 months. The PVI timing was based on the time of referral, not the time of the first AF diagnosis (DAT), as in our study. The study did not report the DAT in the groups. DAT is an evolving indicator of the status of atrial substrate and electrical and structural remodeling that progress during the AF course [5,23]. Missing such a parameter limits the ability to evaluate the procedural outcomes. Moreover, the early-ablation group included patients who had a history of stroke and a higher CHA2DS2VASc score. This could partially explain the lack of difference in ablation outcomes between the groups. In addition, the study did not report the impact of DAT on cardiovascular hospitalizations or AF progression.

### 4.2. Early RFCA and Secondary Effectiveness Outcomes

The early-RFCA group had significantly fewer cardiovascular hospitalizations compared to the late-RFCA group at a median follow-up of 16 months. Kawaji et al. found the same finding at a longer 5-year follow-up [18]. These findings are in line with the EAST—AFNET 4 trial [24], which supports the initiation of a rhythm-control strategy over a rate-control strategy in reducing hard cardiovascular endpoints (composite risk of cardiovascular death, stroke, or hospitalization for worsening HF or acute coronary syndrome) when applied early (<1 year) during the AF course after a median of 5.1 years of follow-up. However, patients in our cohort were younger and with lower stroke risk (59.15 ± 10.7 years and mean CHA2DS2VASc score 1.38 ± 1.1 in our study vs. 70.2 ± 8.4 years and mean CHA2DS2VASc score 3.4 ± 1.3 in the EAST—AFNET 4 trial). Furthermore, patients who receive RFCA within one year after AF diagnosis are less likely to experience worsening of their medical condition or complications (heart failure, arrhythmia) that may require hospitalizations. In addition, the higher rate of AAD use after ablation might be attributable to longer DAT and higher symptom burden post-ablation (as evident by higher ED visits) in the late-RFCA group.

Repeat RFCA was performed in 21 patients (16.2% of all patients). Reconnected pulmonary veins were found in all patients who underwent repeat RFCA. Three patients in the late-RFCA group also experienced left atrial flutter, which could be attributed to the advanced stage of atrial electrical and structural remodeling among these patients.

Despite being underreported in the literature, the rate of AF progression following CA is an important parameter in the management of AF disease. AF progression is altered by CA, either as an initial rhythm control strategy [25] or after attempts of failed AAD [26]. Interestingly, our study reveals that AF progression was not reported when RFCA was conducted early, but did occur when RFCA was performed more than a year after AF diagnosis. To the best of our knowledge, our study is the first to evaluate the impact of PVI timing on the rate of AF progression in a group of young AF patients with relatively low stroke risk. The 2023 American Heart Association (AHA)–American College of Cardiology (ACC)–Heart Rhythm Society (HRS) AF guidelines recommend performing CA in this group of patients (younger patients with few comorbidities) not only to improve symptoms but also to reduce progression [27]. Wijffels et al. described the “AF begets AF” concept, illustrating potential mechanisms that promote AF initiation, maintenance, and progression [28]. Shortening, spatial dispersion of atrial refractoriness, ion channel dysfunction and slowing of intra-atrial conduction induced by AF itself are major modulators linked to the atrial substrate that promote AF remodeling and fibrosis [28]. Therefore, early restoration and maintenance of sinus rhythm may represent a plausible explanation of the substantially low rate of AF progression in the early-RFCA group in our study. Consequently, initiating and intensifying an effective rhythm-control strategy via early RFCA is crucial to break the vicious cycle of AF progression, especially in younger AF patients with few comorbidities.

### 4.3. Early RCFA and Safety Outcomes

Despite the growing evidence in favor of adopting an early-ablation strategy during the course of AF disease [2,5], translation into clinical practice may encounter a major concern regarding patient safety. Procedure-related complications should always be considered [8]. We found two small arteriovenous fistulae (1.5%, one fistula in each group), which were managed conservatively. The rate of such complications is comparable to the reported rate (~1%) in a worldwide survey on the safety of CA of AF [29]. In our study, neither mortality nor serious adverse events such as esophageal fistulae or cardiac tamponade were reported.

### 4.4. DAT as a Predictor of Atrial Arrhythmia Recurrence after Single RFCA

Our study emphasizes the significance of performing PVI early during the AF course, as DAT of more than one year is the only pre-procedural predictor of atrial arrhythmia recurrence. The more frequent and longer the AF episodes, the more cumulative atrial remodeling. These atrial structural and electrical abnormalities render the CA ineffective [5]. In addition, our findings are consistent with an umbrella review of meta-analyses on the predictors of AF recurrence after CA [30]. Of the 28 associations studied between CA and the risk of arrhythmia recurrence, only a DAT of over a year provided highly suggestive evidence [30]. This lends credence to AF being a key driver of atrial remodeling; thus, early RFCA could improve procedural outcomes and halt AF progression.

### 4.5. Clinical Implications

Our findings may provide valuable insights to optimize patient care by improving clinical practice. Attempts to use more than one AAD should not delay the decision of referral for RFCA. Among the known predictors of worse AF ablation outcomes, such as persistent AF, time to RFCA is a prompt, convenient, and easily adjustable predictor to improve ablation outcomes. Moreover, data-driven approaches have the potential to improve health-care delivery by making it more efficient and effective.

## 5. Limitations

Different limitations should be addressed. Firstly, our study enrolled a group of relatively young AF patients with few comorbidities. Therefore, our findings should be interpreted with caution when considering older AF patients with multiple comorbidities. Secondly, it is important to acknowledge that administration of mineralocorticoid receptor agonists (MRAs) and sodium–glucose cotransporter 2 (SGLT2) inhibitors lowers the risk of atrial arrhythmia recurrence following AF ablation [31,32]. These beneficial effects received less attention in our analysis. Thirdly, less than 10% of patients had ICMs that are known to provide more accurate detection of AF recurrences, particularly asymptomatic episodes. Nonetheless, the proportion of ICM monitoring did not differ between the groups and was unlikely to affect the findings of our study. Additionally, unmeasured variables (such as patient preference, COVID infection, the need for COVID vaccination, and regional access to ablation-capable centers) could not be excluded and might influence DAT. Despite this, our study suggests that DAT is a reliable predictor of ablation outcomes. Lastly, this study did not incorporate LA parameters using cardiac magnetic resonance or speckle tracking echocardiography. Such advanced imaging modalities can provide valuable data regarding LA function and fibrosis [7] that may influence AF recurrence and progression.

## 6. Conclusions

Early RFCA within one year of AF diagnosis is associated with less atrial arrhythmia recurrence, cardiovascular hospitalizations, AF progression, and AAD use. Longer DAT (>1 year) and AF recurrence during the blanking period are independent predictors of atrial tachyarrhythmia recurrence after single RFCA.

## Data Availability

Data are available upon reasonable request.
